# Plumbagin inhibits proliferation and promotes apoptosis of ovarian granulosa cells in polycystic ovary syndrome by inactivating PI3K/Akt/mTOR pathway

**DOI:** 10.1080/19768354.2020.1790416

**Published:** 2020-07-17

**Authors:** Zhaowei Cai, Shaojuan He, Tao Li, Li Zhao, Kerong Zhang

**Affiliations:** a Reproductive Center, SSL Central Hospital of Dongguan City, Dongguan City, People’s Republic of China; b Department of Clinical Laboratory, Dongguan People’s Hospital, Dongguan City, People’s Republic of China; c Guangdong Provincial Key Laboratory of Medical Molecular Diagnostics, Guangdong Medical University, Dongguan City, People’s Republic of China; d Department of Gynaecology and Obstetrics and Reproductive Medicine, Second Clinical Medical College of Guangdong Medical, Guangdong Medical University, Dongguan City, People’s Republic of China

**Keywords:** PI3K/Akt/mTOR signal pathway, polycystic ovary syndrome, plumbagin, apoptosis, ovarian granulosa cells

## Abstract

Polycystic ovary syndrome (PCOS) is recognized as a general endocrine disease and reproductive disorder. Although evidence indicates that PCOS has a complex etiology and genetic basis, the pathogenic mechanisms and signal pathway in PCOS remain unclear. In this study, the normal structure of follicle and corpus luteum were observed, and no cyst nor hyperemia was observed under the light microscopic study with hematoxylin and eosin (H&E) staining. Eestosterone and progesterone were evaluated by radioimmunoassay in rat serum. The alterations of proliferative ability and cell cycle distribution of each group were assessed by Cell Counting Kit-8 (CCK8) assay and flow cytometry. The protein expression of p-mTOR/mTOR, p-PI3K/PI3K, p-AKT/AKT, and GAPDH were analyzed by western blotting. Both doses of PLB could benefit the ovarian morphology and polycystic property. PLBinduced a suppress effect on the proliferation of rat ovarian granulosa cells. In addition, PLB also induced concentration-dependent apoptosis in rat ovarian granulosa cells. The rat ovarian granulosa cells treated with PLB that the expression levels of p-AKT, p-mTOR, and p-PI3K were significantly decreased in a concentration-dependent manner. PLB not only plays a critical role in attenuating the pathology and polycystic property changes in the ovary but can also induce rat ovarian granulosa cell apoptosis through the PI3K/Akt/mTOR signal pathway. This study showed the innovative role of PLB in the pathogenesis of PCOS and provides a new therapeutic modality for the treatment of PCOS.

## Introduction

Polycystic ovary syndrome (PCOS) is one of the most general metabolic and endocrine diseases among women of reproductive age (Carmina and Lobo 1999; Glintborg and Andersen 2010). Its pathogenesis is extremely complicated and most frequently characterized by hyperandrogenic (Azziz et al. 2006), chronic anovulation, polycystic ovaries (Brettenthaler et al. 2004), and ovulatory dysfunction (Dewailly et al. 2011). The patients with PCOS are susceptible to reproductive abnormalities and metabolic syndrome, leading to upgraded the risk of developing the cardiovascular disease (Huang and Coviello 2012). Therefore, PCOS is a challenge for clinical and basic research. Although evidence indicates that PCOS has a complex etiology and genetic basis, the pathogenic mechanisms and signal pathway in PCOS remain unclear. Several studies have reported that 90–100% of women with PCOS have aberrational of folliculogenesis and polycystic ovaries with more follicles than healthy ones (Franks et al. 2006). The follicle number, testosterone level in serum and androstenedione concentrations have a positive correlation in PCOS women (Georgopoulos et al. 2014; Stanosz et al. 2018). In PCOS, granulosa cells are also selectively insulin resistant and can lead to hyperinsulinemia and alter intraovarian paracrine signaling to disrupt follicle growth (Giudice 1992; Belani et al. 2018). This condition causes menstrual irregularity and the small antral follicles disorder within the ovary, lead to a polycystic morphology (Goodarzi et al. 2011). The PI3K/AKT/mTOR signaling pathway performs closely and pivotally regulates ovarian functions, including the stability of primordial follicles and the differentiation of granulosa cell (Bai et al. 2015). The PI3K/AKT/mTOR pathway is highly expressed in patients with ovarian cancer (Altomare et al. 2004). Therefore, regulation of the proliferation or apoptosis of granulosa cells in follicles probably is the direction of PCOS therapy.

Plumbagin (PLB; 5-hydroxy-2-methyl-1,4-naphthoquinone) is one of the simplest plant secondary metabolites extracted from the *Plumbago zeylanica* L roots (Gangopadhyay et al. 2008); they exhibit pharmacological effect, such as antiinflammatory, antioxidant, anticancer, and antibacterial (Hwang et al. 2015). Recent studies have shown that PLB exhibited high cytotoxicity in numerous cancer cell lines and induced DNA base damages through increased intracellular reactive oxygen species (ROS) production. Furthermore, PLB exhibits the effect of anticancer therapy by a mechanism that involves autophagic cell death (Li et al. 2014). Accordingly, we investigated whether PLB regulates the apoptosis or cell viability of granulosa cells through the PI3K/Akt/mTOR pathway in POCS.

## Methods

### Animals and PCOS rat model development and treatment

Spragne-Dauley (SD) female rats (body weight 200–250 g, 8-week-old) were purchased from Shanghai Slack laboratory animal co. All animal experiments were approved by the Ethics Committee of SSL Central Hospital of Dongguan City for the use of animals and conducted in accordance with the National Institutes of Health Laboratory Animal Care and Use Guidelines (2011). Animals were conditioned under standard laboratory conditions and housed in 12 h light/12 h dark cyclical alternates and temperature-controlled environment. The animals were randomly separated into three groups, the rats in PCOS group (*n* = 6) were injected hypodermically dehydroepiandrosterone (DHEA, Sigma, USA) 6 mg/kg/day for 20 days, the rats in PCOS + PLB group (*n* = 6) were injected hypodermically dehydroepiandrosterone (DHEA) 6 mg/kg/day and PLB 10 mg or 50 mg/100 g per day for 20 days, the rats in the control group (*n* = 6) were subcutaneous solvent in the same period.

### Primary granulosa cell cultures

The primary granulosa cells were isolated from 45-day-old SD female rats and cultured to examine the mechanism of PCOS. Granulosa cells were cultured in 12-well culture plates with DMEM containing 10% bovine serum under 5% CO_2_ at 37°C and grown to 80–90% confluence. Before the experiment, cells were serum starved for 24 h. Cell viability was examined by trypan blue exclusion method.

### Hormone assays

In experiments to quantify hormone secretion, the abdominal aortic blood was collected from each group animals anesthetized by 3.5% chloral hydrate (350 mg/kg body mass). The whole blood from each group of animals was centrifuged for 15 min (2500 g) and the serum was separated, after being left for 2 h at room temperature. Finally, the concentrations of testosterone and progesterone in serum were detected by Radioimmunoassay kits.

### Hematoxylin and eosin staining analysis

The collected ovaries were fixed in a 10% formaldehyde solution. The dehydrated tissues were sectioned at 5 μm slices; every 10th section (*n* = 6) was mounted on a glass slide and stained with H & E. Place a drop of Permount over the tissue on each slide and add a coverslip. View the slides and measured the sections under a microscope.

### Western blot analysis

Protein was lysed and extracted from cultured primary granulosa cells. Protein extract was separated by 10% SDS-PAGE and transferred to anitrocellulose membrane. After being blocked with 5% nonfat milk, the membranes were immersed using primary antibodies (anti-PI3K (ab86714, Abcam), anti-pPI3K (sc-1637, Santa Cruz Biotechnology), anti-AKT (ab8805, Abcam), anti-pAKT (sc-7985-R, Santa Cruz Biotechnology), anti-mTOR (ab2732, Abcam), anti-pmTOR (ab84400, Abcam), anti-GAPDH (ab9485, Abcam)) at 4°C overnight. Next, the membranes were incubated with 1:3000 dilution secondary antibodies for 1 h. The specific protein bands were detected with the enhanced chemiluminescence (ECL) system. Density of each band was measured by Image J.

### Flow cytometry

The primary rat ovarian granulosa cells were collected and some cells were added with 100 µl propidium iodide (PI)-Rnase A for 15-min incubation without light exposure as control, the cell cycle was measured by flow cytometry. The percentages of apoptosis in primary rat ovarian granulosa cells were measured using annexin-v. Cells in each group were staining with a combination of annexin-v-fluorescein isothiocyanate (FITC) and PI, then blended and incubated for 15 min, the number of apoptosis cells was quantiﬁed by flow cytometry.

### CCK8 assay

The primary rat ovarian granulosa cells were suspension (100 μl/well) in 96-well plate. After treating different concentration of PLB for 24 h, 10 μl CCK-8 reagent were added into cells in each well for incubation at 37°C for 3 h. Each group was detected and measured the absorbance at 450 nm using a microplate reader (Bio-Rad, Hercules, CA, USA). Cell viability rate = (OD experimental group/OD blank control group) × 100%.

### Statistical analysis

Statistical analyses were conducted using SPSS 21.0 software (Armonk, NY, USA). All quantitative data were expressed as mean ± standard deviation. Normality tests were tested by one-way analysis of variance (ANOVA). Tukey’s multiple comparisons test was used for pairwise comparisons after the ANOVA. *p* < .05 was considered a statistically significant difference.

## Results

### Plumbagin attenuates the pathology and polycystic property changes of the ovary in polycystic ovary syndrome rats

**Figure 1.**
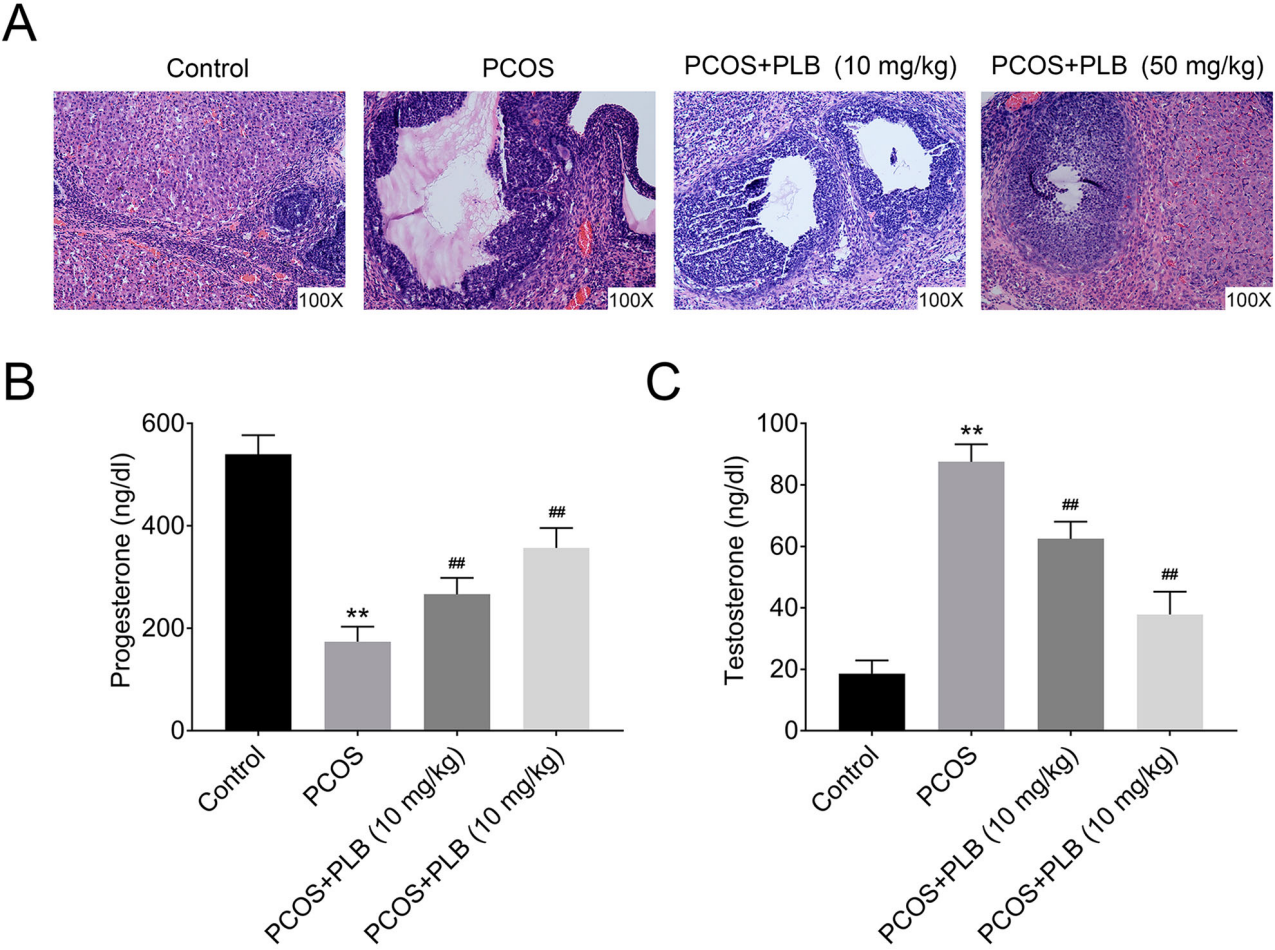
The effect of PLB on ovarian in PCOS rats. **(A)** The effect of PLB on ovarian histopathology in normal and PCOS rats, determined by H&E staining. **(B)** The progesterone expression level in normal and PCOS rats, determined by radioimmunoassay. **(C)** The testosterone expression level in normal and PCOS rats, determined by radioimmunoassay. Statistical analysis was performed according to Material and Methods (Error Bar: Mean ± SD, **p < .01 compared to control group, and ##*p *< .01 compared to PCOS group).

In the ovary of control group, the normal structure of follicle, mature oocytes, and corpus luteum were observed, and no cyst nor hyperemia was observed under the light microscopic study with H&E staining. In the group with PCOS induced by DHEA, pathological examination showed a disordered ovarian structure and increased hemorrhagic, hyperemia and cystic follicles. Moreover, the granular cell layers were reduced and sparsely arranged, and luteal formation significantly decreased in the majority of ovarian follicles (Figure 1(A)). However, the continuous treatment by 10–50 mg/ml PLB in PCOS rats showed recovery and attenuated polycystic features, as demonstrated by microscopic morphological examination. The microscopic study with H&E staining in PCOS + PLB groups showed that the rate of these pathological changes in DHEA treatment decreased, the cysts significantly reduced, and endometrial tissue showed better arrangements than the PCOS group (Figure 1(A)).

Compared with the control group, the serum concentrations of progesterone in the PCOS group significantly decreased (Figure 1(B)). The serum levels of testosterone were significantly higher in the PCOS group (Figure 1(C)). The intervention with increased concentrations of PLB (10 and 50 mg/ml) induced a dose-dependent increase in progesterone in PCOS + PLB groups (Figure 1(B)). In addition, the serum levels of testosterone significantly decreased in both PCOS + PLB groups relevant to the PCOS group (Figure 1(C)). These results indicate that both doses of PLB (10 and 50 mg/ml) could benefit the ovarian morphology and polycystic property.

### Plumbagin inhibits the proliferation of rat ovarian granulosa cells

**Figure 2.**
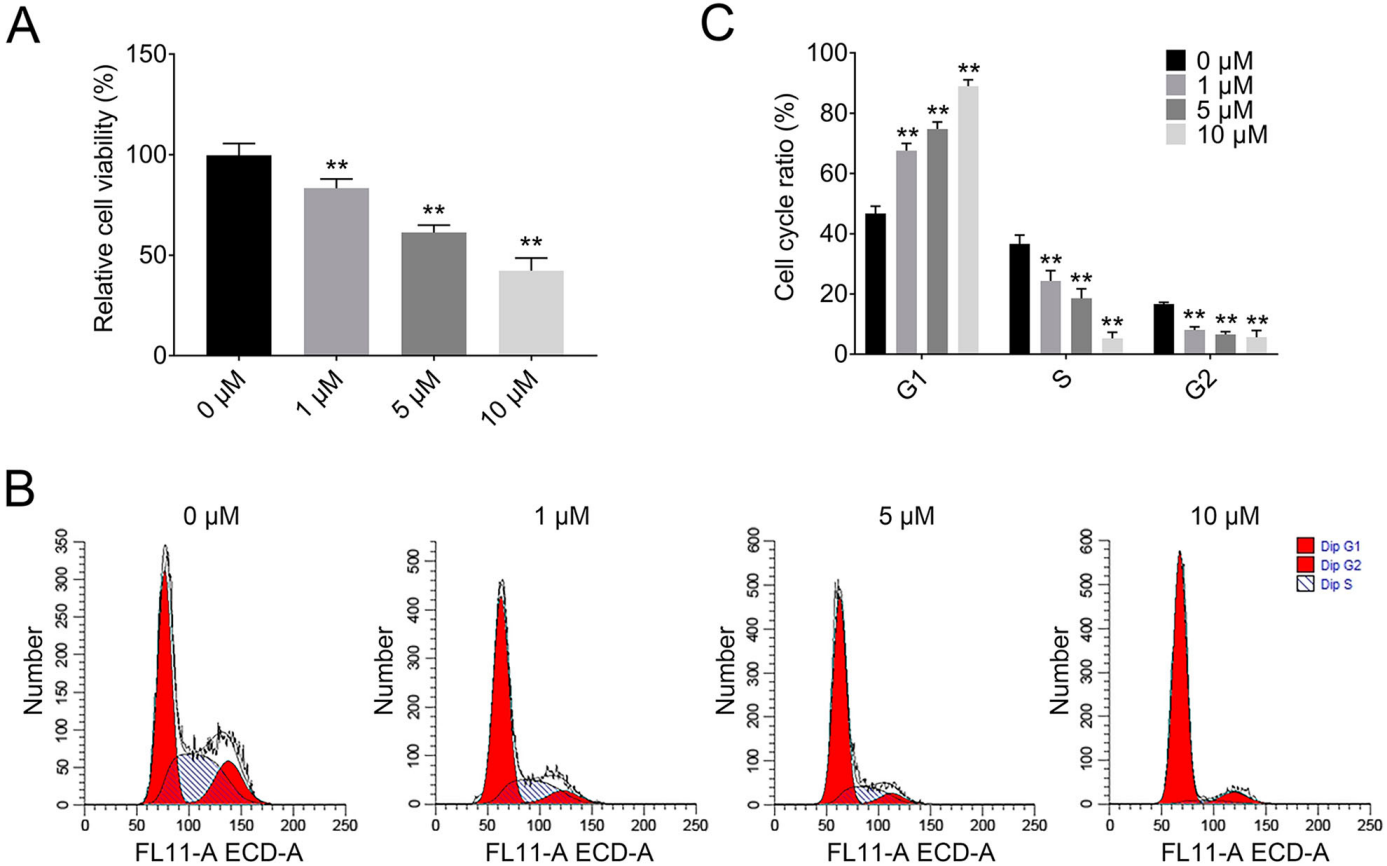
PLB inhibits the proliferation of rat ovarian granulosa cells in PCOS. **(A)** The relative cell viability level in rat ovarian granulosa cells treated with PLB at 0, 1, 5 and 10 µM, determined by CCK8 assay. **(B)** Cell cycle distribution of rat ovarian granulosa cells treated with PLB at different concentration was detected by flow cytometry. **(C)** Bar graphs showing the ratio of cell cycle in rat ovarian granulosa cells treated with PLB at 0, 1, 5 and 10 µM for 24 h. Statistical analysis was performed according to Material and Methods (Error Bar: Mean ± SD, **p < .01).

The primary granulosa cells were collected from 45-day-old rats and cultured to investigate the mechanism of PCOS. CCK8 assay and flow cytometry were executed to assessed the variation in proliferative ability and determine the cell cycle distribution of each group. Based on the aforementioned results, to investigate the effect of PLB on potential growth inhibition of ovarian granulosa cells, we treated the rat ovarian granulosa cells with different concentrations of PLB for 24 h. The relative cell viability of each group was determined by CCK8 assay. The results showed that when rat ovarian granulosa cells were treated with PLB at 1, 5, and 10 µM for 24 h, the relative cell viability of rat ovarian granulosa cells declined (Figure 2(A)). The effect of increasing concentrations of PLB (1, 5, and 10 µM) on cell cycle distribution was determined by flow cytometry. The ratio of rat ovarian granulosa cells in G0/G1 phase increased, and the ratio of cells in S and G2/M phases reduced (Figure 2(B) and (C)). These results demonstrate that PLB induced an inhibitory effect on the proliferation of rat ovarian granulosa cells.

### Plumbagin induces the apoptosis of rat ovarian granulosa cells

**Figure 3.**
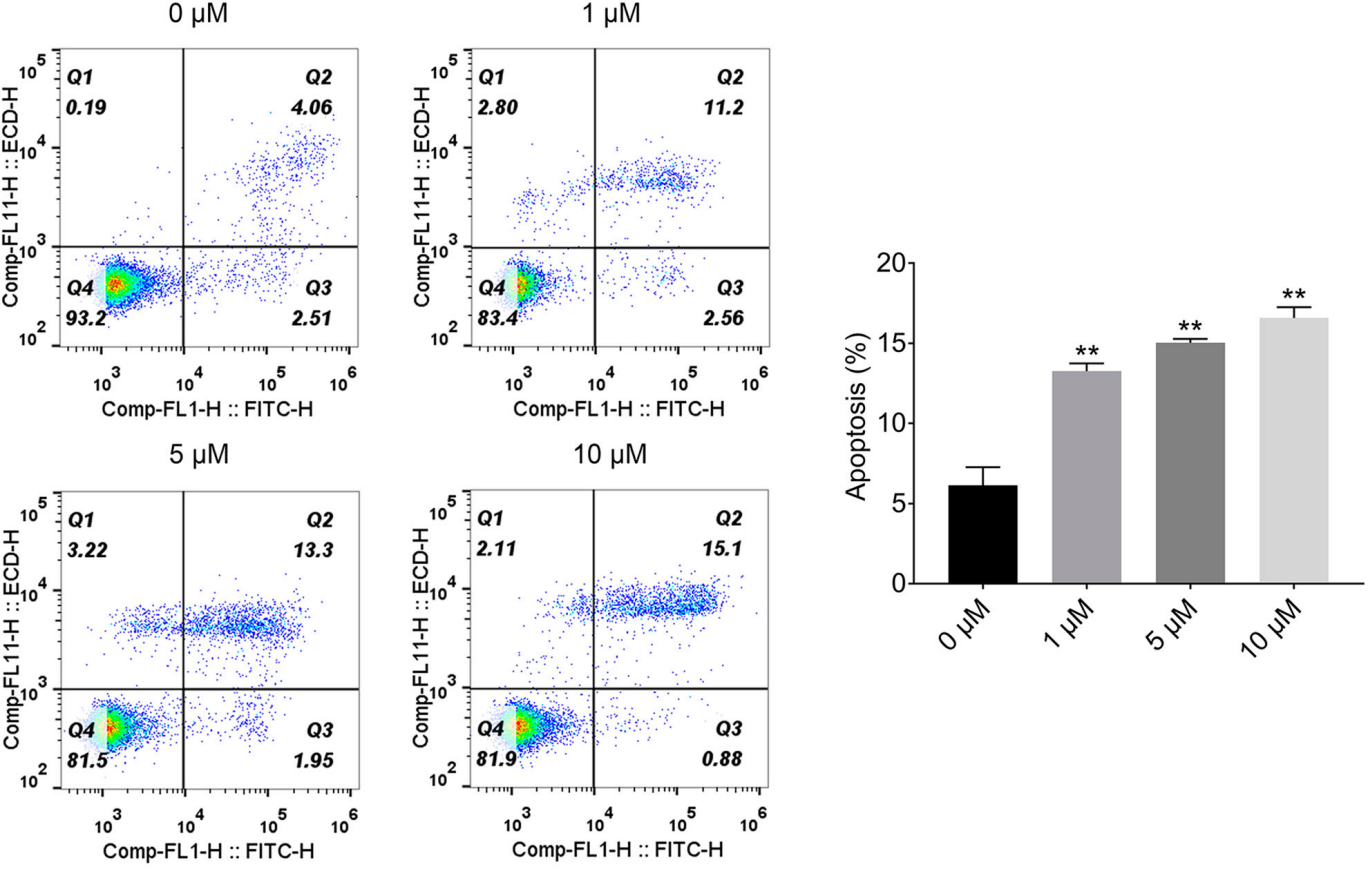
PLB induces the apoptosis of rat ovarian granulosa cells in PCOS. Apoptosis of rat ovarian granulosa cells was measured by flow cytometric analysis. The bar graphs showing the ratio of apoptosis cells in each groups. Statistical analysis was performed according to Material and Methods (Error Bar: Mean ± SD, ** *p *< .01).

To determine the PLB effect on apoptosis of rat ovarian granulosa cells, we treated the primary culture of rat ovarian granulosa cells with different concentrations of PLB for 24 h and quantified the number of apoptotic cells by flow cytometric analysis. As shown in Figure 3, a small amount of apoptotic cells (5 −7.0%) could be found in the control group (0 µM), whereas rat ovarian granulosa cells treated with PLB at 1, 5, and 100 µM for 24 h yielded a large number of apoptotic cells (early + late apoptosis). These results demonstrate that PLB induced cellular apoptosis in rat ovarian granulosa cells.

### Plumbagin induces apoptosis in rat ovarian granulosa cells via inhibition of PI3K/AKT/mTOR pathway

**Figure 4.**
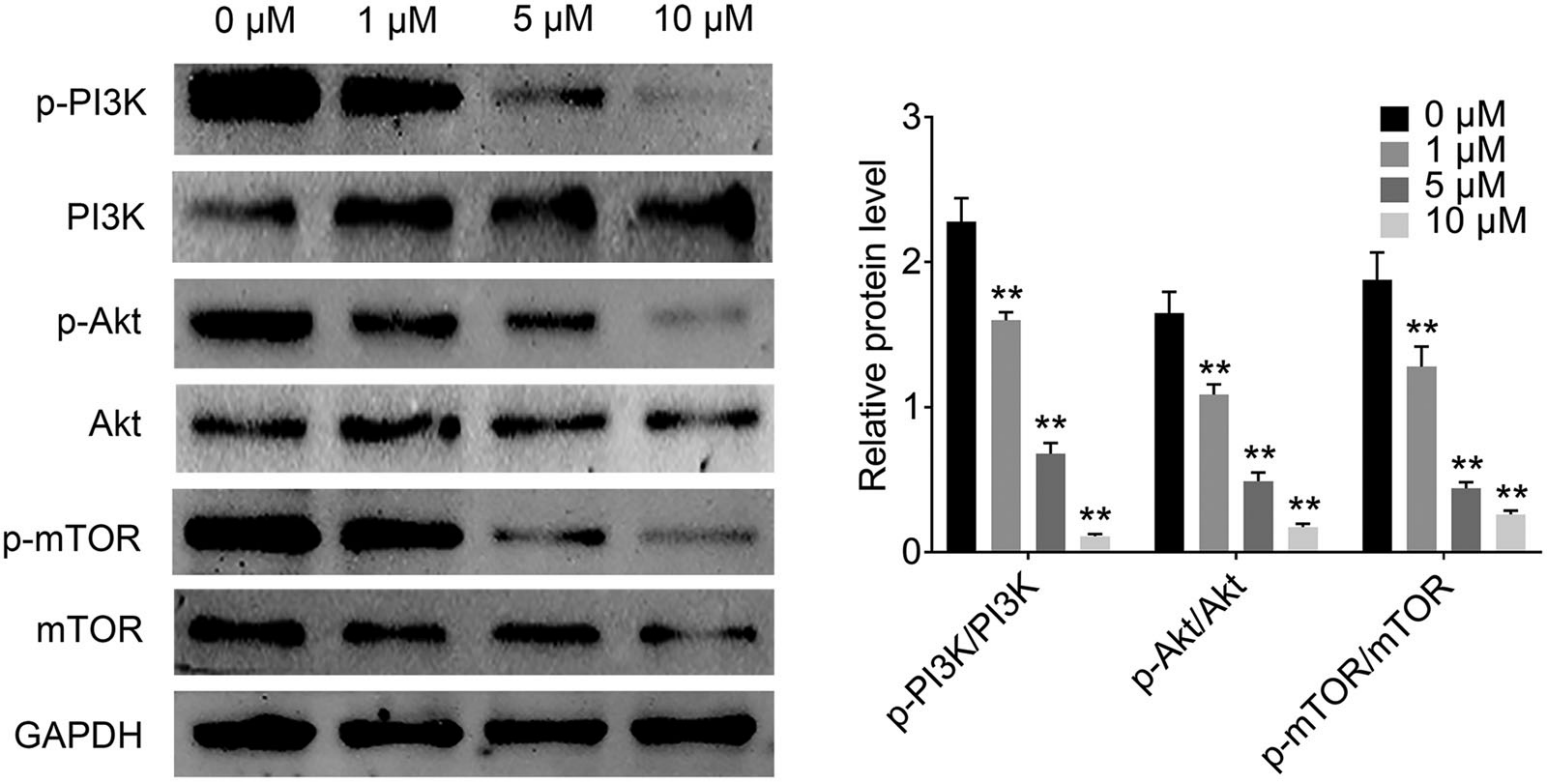
PLB induces the apoptosis of rat ovarian granulosa cells in PCOS via PI3K/Akt/mTOR signal pathway. Immunoblotting for PI3K, Akt and mTOR after different concentration of PLB treatment in rat ovarian granulosa cells. The bar graphs showing the relative protein level of PI3K, Akt and mTOR in each groups. Statistical analysis was performed according to Material and Methods (Error Bar: Mean ± SD, ** *p *< .01).

PCOS is a complex multifactorial endocrinopathy that is characterized by granulosa cell dysfunction, various metabolic derangements, and insulin resistance. The AKT signaling pathway is important in controlling growth factors, hormones, and cellular functions, including metabolism and cell proliferation. To determine whether PI3K/AKT/mTOR expression is involved in the pathogenesis of PCOS, we compared the p-AKT, p-mTOR, and p-PI3K expressions between PCOS and PLB treatment groups via Western blotting of rat ovarian granulosa cells. As shown in Figure 4, the protein levels of p-AKT, p-mTOR, and p-PI3K significantly increased in the non-PLB treatment group. The rat ovarian granulosa cells were treated with PLB at 1, 5, and 10 µM for 24 h, and the expression levels of p-AKT, p-mTOR, and p-PI3K significantly decreased in a concentration-dependent manner (Figure 4). However, the mRNA levels of AKT, mTOR, and PI3K have no significant difference (Supplementary Figure 1). The mRNA levels of CyclinD1 and Bcl2 were significantly decreased in a concentration-dependent manner, conversely, a significant dose-dependent increase was shown in the mRNA level of Bax (Supplementary Figure 1). The results indicate that PLB not only plays a critical role in attenuating the pathology and polycystic property changes in the ovary but can also induce rat ovarian granulosa cell apoptosis via the PI3K/AKT/mTOR signal pathway.

## Discussion

PCOS is recognized as a general endocrine disease and reproductive disorder with three key diagnostic features, including polycystic ovaries, hyperandrogenism, and chronic anovulation (Hart et al. 2004). PCOS is usually associated with insulin resistance, which leads to steroidogenesis lipogenesis and abnormal metabolism of lipids (Hart et al. 2004). Recent studies have indicated that the abnormal expression of the PI3K/Akt/mTOR signal pathway can impair various ovarian functions, induce the risk of insulin resistance, and affect the proliferation of follicles (Mabuchi et al. 2015). The suppression of insulin secretion may reverse the ovarian function and decrease androgen levels in PCOS (De Leo et al. 2003). PLB has been reported to affect multiple signaling pathways and exhibit anticancer activities by inducing ROS generation in cancer cells (Sagar et al. 2014). However, the mechanism of PLB in PCOS and the interaction with the PI3K/Akt/mTOR signaling pathway in the pathogenesis of ovarian diseases remain unclear.

In this study, we explored the effect of PLB on pathology and polycystic property changes in ovaries of rats treated with DHEA to mimic PCOS. The DHEA-treated rats revealed different sizes of follicles and compact formation of granulosa cells in the ovarian cortex. Our results showed that the continuous treatment by 10 or 50 mg/ml PLB in PCOS rats showed recovery to attenuated polycystic features. The intervention with increased concentrations of PLB induced a dose-dependent increase in progesterone in the PCOS + PLB groups. Furthermore, the serum levels of testosterone significantly decreased in both PCOS + PLB groups. The results of this study further confirm that PLB could benefit the understanding of ovarian morphology and polycystic property.

A recent study showed that PLB displayed an inhibitory effect on the proliferation and viability of numerous cancer cell lines (Wu et al. 2016). In our study, PLB caused a significant concentration-dependent inhibition of the proliferation of rat ovarian granulosa cells. Moreover, we inspected the increased apoptosis of primary-cultured rat ovarian granulosa cells treated with PLB at 1, 5, and 100 µM for 24 h. These results further confirm the notion that PLB exerts apoptotic and anti-proliferation functions in many cell types.

Numerous studies showed that PCOS is characterized by follicular failure, granulosa cell dysfunction (Hong et al. 2018), hyperinsulinemia, and insulin resistance. Insulin resistance is highly associated with the overexpression of PI3K/Akt/mTOR signal pathway, which is a universal kinase that has been appeared in the control of different growth factors and hormones (Martini et al. 2014). This pathway also regulates cellular functions, including metabolism, growth, and cell proliferation. In our study, the expression levels of p-mTOR, p-AKT, and p-PI3K significantly increased in the PCOS group, and the PLB treatment at 1, 5, and 100 µM for 24 h reversed the effect on rat ovarian granulosa cells. The results of PLB-induced PI3K/Akt/mTOR signal pathway inhibition in rat ovarian granulosa cells are consistent with the findings obtained with other cancer cell lines. This study has confirmed that PLB induces apoptosis of rat ovarian granulosa cells via suppression of the PI3K/Akt/mTOR signal pathway.

In conclusion, PLB not only plays an important role in attenuating the pathology and polycystic property changes in the ovary but can also induce rat ovarian granulosa cell apoptosis via the PI3K/Akt/mTOR signal pathway. This study showed the novel finding of PLB in the pathogenesis of PCOS and provides a new therapeutic modality for the treatment of PCOS.

## Authors’ contributions

ZWC conceived and designed the experiments, SJH and TL analyzed and interpreted the results of the experiments, ZL and KRZ performed the experiments.

## Supplementary Material

Supplemental Material

## Data Availability

All data generated or analyzed during this study are included in this published article.
