# Supplementary figures and images for: Plumbagin inhibits proliferation and promotes apoptosis of ovarian granulosa cells in polycystic ovary syndrome by inactivating PI3K/Akt/mTOR pathway
Source: Anim Cells Syst (Seoul). 2020 Jul 17;24(4):197–204. doi: 10.1080/19768354.2020.1790416 (PMC7473319; doi:10.1080/19768354.2020.1790416)

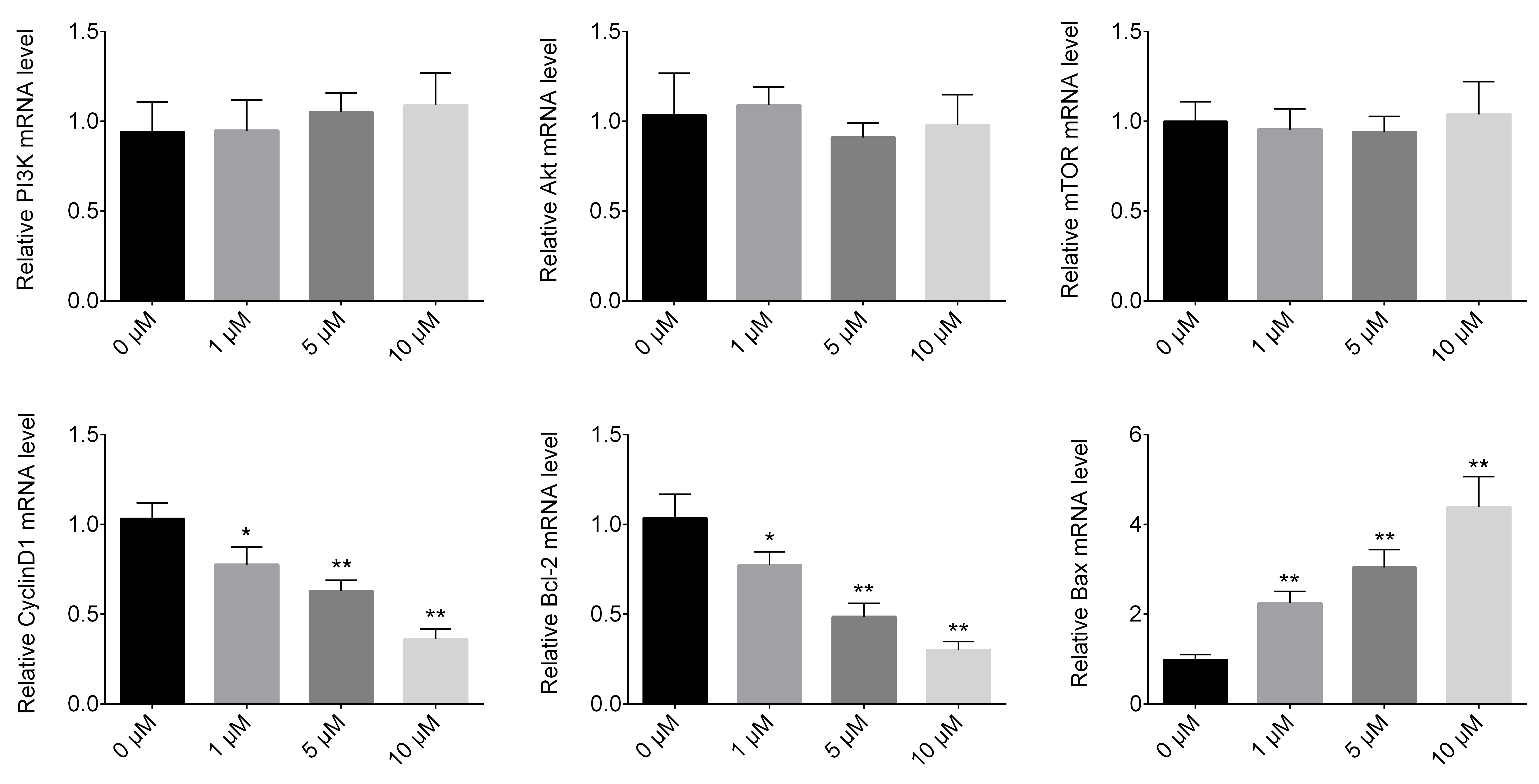

Supplement: Supplemental Material [file TACS_A_1790416_SM8317.jpg]
